# Emerging insights into inflammation-driven atherosclerosis: immune cell mechanisms

**DOI:** 10.3389/fcvm.2026.1839374

**Published:** 2026-07-02

**Authors:** Abdullahi Osman Mohamud, Zhiyin Dai

**Affiliations:** 1 Department of Cardiology, Affiliated Hospital of Jiangsu University, Zhenjiang, Jiangsu Province, China; 2 Institute of Cardiovascular Diseases, Jiangsu University, Zhenjiang, Jiangsu Province, China

**Keywords:** atherosclerosis, clonal haematopoiesis, gut microbiota, meta-inflammation., single-cell omics, trained immunity

## Abstract

Atherosclerosis is a chronic inflammatory disease marked by the deposition of lipids, fibrous components, and calcification in the major arteries. The process is initiated by endothelial activation, which increases vascular permeability, promotes leukocyte adhesion, and leads to the migration of inflammatory cells into the arterial wall. These events trigger vascular constriction and activate inflammatory pathways, together promoting atheromatous plaque development. This review integrates emerging concepts in the immune cascade, detailing how recruited immune cells such as macrophages, T cells, B cells, dendritic cells (DCs), and neutrophils interact to sustain inflammation within developing plaques, as revealed by single-cell omics approaches. In addition, we discuss novel ideas, such as phenotypic switching of vascular smooth muscle cells into macrophage-like foam cells and the systemic effects of clonal haematopoiesis. Finally, we explore how systemic and environmental factors, including gut microbiota, epigenetic changes, hypertension, diabetes, obesity, and smoking, maintain a state of trained immunity and meta-inflammation that exacerbates disease, thus providing a conceptual framework for targeting inflammatory axes in future therapeutic strategies.

## 1 Introduction

Despite decades of lipid-lowering therapy, atherosclerotic cardiovascular disease remains the leading cause of death globally, accounting for approximately 17.9 million deaths annually and imposing a substantial health and economic burden worldwide (1, 2). Atherosclerosis underlies most CVD cases, giving rise to coronary artery disease (CAD), ischemic stroke, and peripheral arterial disease (3). Once regarded solely as a disorder of lipid accumulation within vessel walls, atherosclerosis is now recognized as a chronic inflammatory and immune-mediated disease in which both innate and adaptive immunity are active at every stage of plaque development (4–6).

Recent advances in high-resolution profiling technologies have transformed our understanding of this complexity. Single-cell RNA sequencing (scRNA-seq) and spatial transcriptomics have revealed substantial heterogeneity among immune cells within atherosclerotic plaques, uncovering subsets with distinct and sometimes opposing functional roles that cannot be captured by conventional classification systems. Furthermore, the discovery of clonal haematopoiesis of indeterminate potential (CHIP) has established a mechanistic link between age-related somatic mutations in hematopoietic stem cells, amplified vascular inflammation, and elevated cardiovascular risk (7, 8).

While prior reviews have addressed canonical immune pathways in atherosclerosis, none have comprehensively integrated emerging single-cell and spatial transcriptomic data alongside systemic modulators, including epigenetic reprogramming, trained immunity, and gut microbiota, within a unified framework. This review addresses that gap. We examine the contributions of major immune cell types, including macrophages, dendritic cells, neutrophils, and T and B lymphocytes, as characterized by single-cell technologies, the systemic and environmental factors that sustain chronic vascular inflammation, and the therapeutic strategies that these insights are beginning to unlock.

## 2 Pathophysiology of atherosclerosis

**Figure 1:**
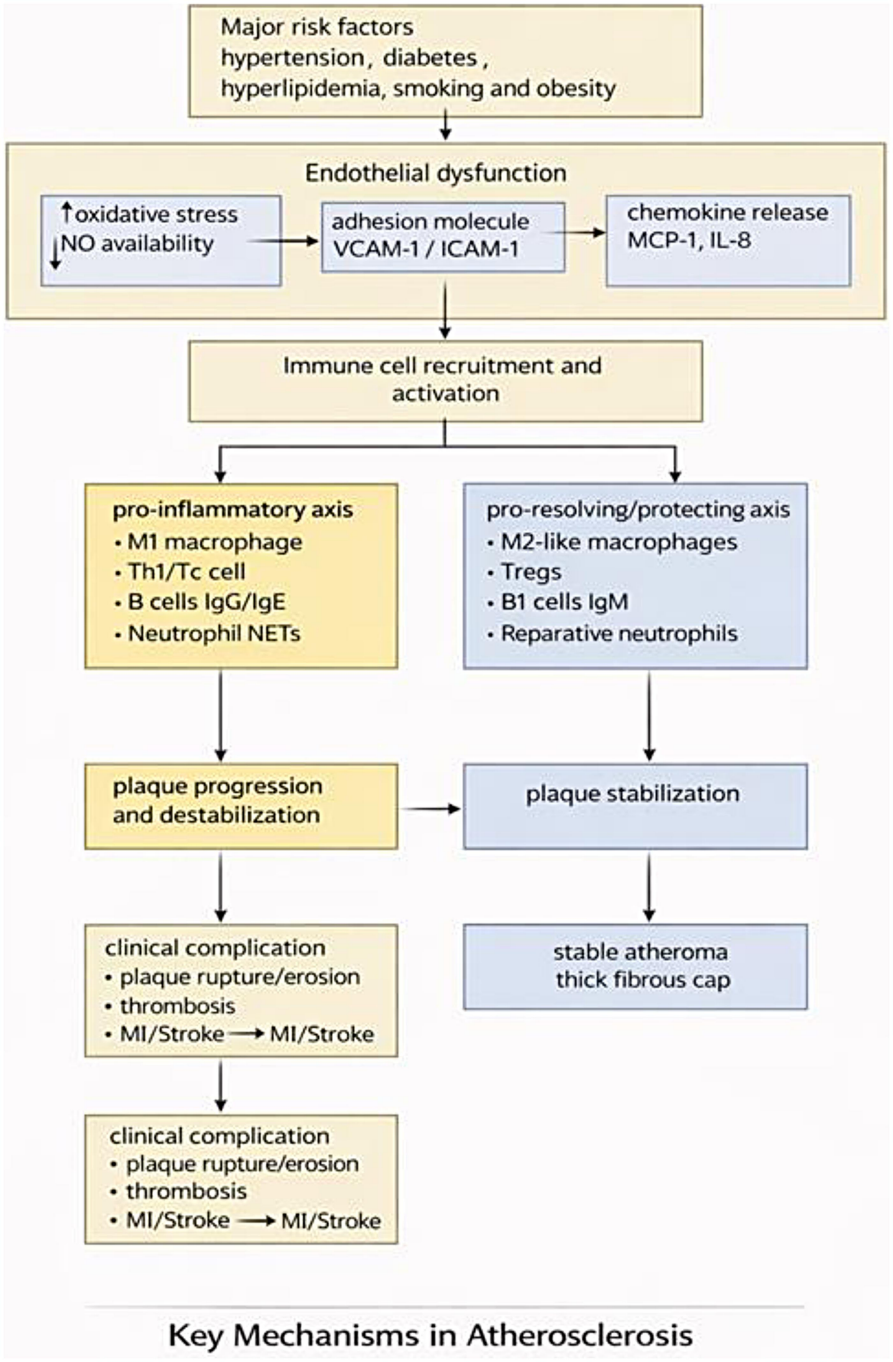
Key mechanisms in atherosclerosis. Created with BioRender.com. in https://BioRender.com.

Major cardiovascular risk factors, including hypertension, diabetes, hyperlipidaemia, smoking, and obesity, initiate endothelial dysfunction, characterized by increased oxidative stress, reduced nitric oxide (NO) bioavailability, upregulation of adhesion molecules such as VCAM-1 and ICAM-1, and release of chemokines including MCP-1 and IL-8. These changes promote recruitment and activation of immune cells within the arterial wall. The inflammatory response then diverges into two opposing pathways: a pro-inflammator*y* axis, involving M1 macrophages, Th1/Tc cells, IgG/IgE-producing B cells, and neutrophil extracellular traps (NETs), which drives plaque progression and destabilization, ultimately leading to plaque rupture or erosion, thrombosis, myocardial infarction (MI), and stroke, and a pro-resolving/protective axis, involving M2-like macrophages, regulatory T cells (Tregs), IgM-producing B1 cells, and reparative neutrophils, which promotes plaque stabilization and formation of a stable atheroma with a thick fibrous cap (Figure 1).

### 2.1 Endothelial dysfunction and activation

Endothelial cells (ECs) are a group of cells that perform many critical functions in the body (7). ECs also help the body fight infections through both innate (immediate, non-specific immunity) and adaptive (specific, learned immunity) mechanisms. ECs are among the first cells in the bloodstream to detect danger signals, such as those from external pathogens (harmful microorganisms) and endogenous metabolites (molecules produced within the body). They serve as danger signal sensors (9–11). The vascular endothelium, which consists of this layer of ECs, acts as a major barrier between flowing blood and tissues (9).

Endothelial dysfunction is a major cause of cardiovascular diseases. It occurs when oxidative stress, metabolic issues, and adipokine dysregulation interact in complex ways (12, 13). When there are excessive reactive oxygen species (ROS), endothelial nitric oxide synthase (eNOS) becomes uncoupled, reducing nitric oxide bioavailability (14). This impairs vasodilation, promotes vascular stiffness, and contributes to a pro-inflammatory endothelial state. Adipokines such as leptin, adiponectin, and resistin exert opposing effects on vascular homeostasis and further modulate inflammation and oxidative stress in obesity and metabolic syndrome (15). Dyslipidaemia, particularly through the accumulation of oxidized low-density lipoprotein (ox-LDL), further drives endothelial injury and accelerates atherosclerosis. ox-LDL acts as a damage-associated molecular pattern (DAMP) and binds receptors such as cluster of differentiation 36 (CD36), oxidized low-density lipoprotein receptor-1 (LOX-1), and Toll-like receptor 4 (TLR4), triggering innate immune activation. CD36 mediates ox-LDL uptake leading to foam cell formation and activates NF-*κ*B signalling, increasing pro-inflammatory cytokines and adhesion molecules. Lectin-like LOX-1 amplifies oxidative stress via ROS produced by the nicotinamide adenine dinucleotide phosphate (NADPH) oxidase, worsening eNOS dysfunction and endothelial damage. In parallel, lipid overload activates the NOD-, LRR-, and pyrin domain-containing protein 3 (NLRP3) inflammasome, promoting interleukin 1*β* (IL-1β) and IL-18 release and pyroptotic cell death, thereby reinforcing vascular inflammation and plaque progression (16, 17).

Studies show that the endothelium, the thin layer of cells lining blood vessels, is where mesenchymal cells associated with plaque originate. Endothelial cells can undergo a process called endothelial-mesenchymal transition (EndMT), in which they stop expressing markers and performing functions typical of endothelial cells and begin expressing markers and performing functions typical of mesenchymal cells, which are more flexible and can move into different tissue areas. Additionally, EndMT can cause mesenchymal cells that come from endothelial cells to break apart and enter the underlying tissue (18).

Endothelial dysfunction marks a shift from a quiescent to a defensive phenotype (19). Most cardiovascular risk factors activate endothelial machinery that produces chemokines, cytokines, and adhesion molecules. These molecules engage leukocytes and platelets, directing inflammation to certain tissues to eliminate pathogens (20).

Endothelial dysfunction is closely linked to persistent low-grade inflammation. Cytokines and chemokines play key roles in causing vascular damage and recruiting immune cells (21). Tumour necrosis factor-alpha (TNF-α) and monocyte chemoattractant protein-1 (MCP-1) are major inflammatory mediators. They act both locally and systemically to activate and harm endothelial cells. MCP-1, or Chemokine (CC motif) ligand 2 (CCL2), is a CC chemokine that attracts and stimulates other inflammatory cells, and its key action is to mobilize and recruit cells, such as monocytes/macrophages, and other cytokines to sites of inflammation. This worsens many illnesses (22).

### 2.2 VSMC phenotypic switching and foam cell formation

Vascular smooth muscle cells (VSMCs) constitute the predominant cell type within the media layer of healthy arteries. VSMC phenotypic switching has been regarded as a relatively simple, reversible transition between contractile and synthetic phenotypes in response to vascular injury (23). However, within the atherosclerotic microenvironment, VSMCs undergo complex phenotypic reprogramming in response to disturbed flow and local pathological stimuli, including downregulation of contractile markers, dedifferentiation, migration, and transdifferentiation into alternative phenotypes such as macrophage-like, foam cell-like, mesenchymal stem cell-like, myofibroblast-like, and osteochondrogenic-like states. These changes are important in plaque formation, progression, calcification, and stability (24, 25).

Recent studies have further demonstrated that a subset of pre-existing VSMCs undergoes clonal expansion and acquires diverse phenotypes in atherosclerotic lesions, driven by epigenetic modifications that affect transcription factor access and gene expression dynamics. Nevertheless, the exact functional roles and relative contribution of individual VSMC-derived phenotypes in atherosclerosis remain incompletely understood (26). Despite this incomplete understanding, several specific VSMC-derived phenotypes have been partially characterized, providing valuable insights into disease mechanisms. For example, transcription factor 21 (TCF21) regulates VSMC phenotypic modulation by disrupting the core contractile transcriptional program controlled by the SRF–MYOCD (serum response factor–myocardin) axis. This promotes the transition of differentiated contractile VSMCs into phenotypically modulated fibromyocytes, which contribute to the formation and stabilization of the fibrous cap in atherosclerotic plaques (27).

The process of foam cell formation begins with subendothelial retention and oxidative modification of low-density lipoprotein (LDL), generating ox-LDL, which is subsequently internalized by vascular cells through SRs, such as CD36, Scavenger Receptor Class A Member 1 (SR-A1), and LOX-1 (28). During atherosclerosis, inflammatory mediators stimulate VSMC migration from the media into the intima, where they progressively lose expression of contractile markers, including *α*-smooth muscle actin (*α*-SMA) and smooth muscle myosin heavy chain (SMMHC), while simultaneously acquiring macrophage-like characteristics and upregulating scavenger receptors such as CD36, CD68, CD11b, and Lectin, Galactoside-Binding, Soluble, 3(LGALS3) (29, 30). These receptors facilitate excessive uptake of ox-LDL and intracellular cholesterol accumulation. Free cholesterol is subsequently esterified by acyl-coenzyme A (acyl-CoA): cholesterol acyltransferase 1 (ACAT1) and stored as cytoplasmic lipid droplets, ultimately producing the characteristic foamy morphology (31).

In addition, the differentiation of VSMCs into foam cells is a canonical form of phenotypic modulation. Foam cells are key cellular components of atherosclerotic plaques. Although they were traditionally considered to arise predominantly from macrophages, recent evidence indicates that VSMCs contribute substantially to foam cell formation, accounting for more than half of the foam cells in plaques. The accumulation of lipid-laden foam cells within the arterial wall plays a central role in the initiation and progression of atherosclerotic (AS) lesions, and excessive foam cell accumulation can promote necrotic core formation within atherosclerotic plaques (32).

## 3 Immune mechanisms in atherosclerosis

There are two types of responses in the immune system: innate and adaptive. The innate immune system's ability to mount rapid, general responses makes it the first line of defence against invasive pathogens. The primary cell types of the innate immune system are DCs, monocytes/macrophages, and neutrophils. Scavenger receptors (SRs) and TLRs are pattern recognition receptors that enable innate immune cells to recognize pathogen- and damage-associated molecular patterns. Though slower, adaptive immunity is more focused (33).

T and B cells recognize specific pathogens or antigen epitopes and generate diverse antigen-specific receptors and immunoglobulins, forming much of adaptive immunity. Immune cell changes significantly contribute to atherosclerotic plaque development by promoting plaque formation and growth (Table 1) (34).

**Table 1:** Immune cell contributions to atherosclerosis.

Immune Cell Type	Subtypes	Major Cytokines/Mediators	Net Effect on Disease	References
Monocytes/Macrophages	resident-like macrophages, inflammatory macrophages, and TREM-2^hi^ macrophages	TNF-α, IL-1β, IL-6	Predominantly pro-atherogenic	(124)
Dendritic cells	Conventional DCs (cDCs), Plasmacytoid DCs (pDCs)	IL-12, IFN-α	Amplify adaptive immune responses (pro-atherogenic)	(125)
Neutrophils	Nh0, Nh1, Nh2, and Nh3.	Myeloperoxidase (MPO), elastase	Promote plaque instability and rupture	(126)
T Lymphocytes	Th1, Th2, Th17, Treg, CD8⁺ cytotoxic T cells	IFN-*γ*, IL-17, IL-10	Dual role (protective and pro-atherogenic depending on subtype)	(127)
B Lymphocytes	B-1, B-2 cells	IgM, IgG, IgE	B-1: protective; B-2: pro-atherogenic	(128)

### 3.1 Innate immunity

#### 3.1.1 Monocytes and macrophages

Monocytes are bone marrow–derived circulating leukocytes that migrate into tissues and differentiate into macrophages, where they contribute to innate immune defence and tissue homeostasis (34). Together, monocytes and macrophages constitute major components of the innate immune system, whereas dendritic cells initiate and coordinate adaptive immune responses and are essential for immunologic memory and tolerance. Recent *in vivo* lineage-tracing and developmental studies in mice have further clarified the relationships among monocytes, macrophages, and dendritic cell populations (35).

Traditionally, macrophage activation has been described using the M1/M2 polarization framework. M1 macrophages, also referred to as classically activated macrophages, exhibit pro-inflammatory functions and promote host defence against pathogens, whereas M2 or alternatively activated macrophages are associated with anti-inflammatory responses, tissue remodelling, and repair (36). This model parallels the Th1/Th2 paradigm proposed by Mosmann and Coffman in 1986 for T-helper-cell differentiation. The M1/M2 concept was later introduced by Charlie Mills in 2000 and subsequently expanded by Shivanand Ghosh, becoming a widely adopted framework for categorizing macrophage responses (37, 38). According to this classical hypothesis, macrophages acquire either M1- or M2-like phenotypes depending on local environmental cues and inflammatory stimuli.

However, increasing evidence indicates that this binary classification is overly simplistic. Recently identified macrophage subsets expressing both M1- and M2-associated markers have challenged the adequacy of the traditional polarization model (39). Over the past decade, numerous studies have revised previous concepts regarding macrophage ontogeny and function, leading to the development of more comprehensive frameworks of macrophage biology (40). Notably, arterial and cardiac macrophages appear to maintain themselves independently of circulating monocytes under steady-state conditions, whereas inflammatory stimuli such as myocardial infarction or hyperlipidaemia promote monocyte recruitment and differentiation into tissue macrophages. Beyond their classical phagocytic role, macrophages are now recognized as highly adaptable immune cells present in nearly all organs, continuously sensing environmental changes and displaying remarkable functional diversity through specialized non-canonical activities (41, 42).

Recent advances in single-cell RNA sequencing have further highlighted the extensive heterogeneity of macrophages within atherosclerotic plaques, demonstrating that plaque macrophages span a spectrum of transcriptional and functional states rather than fit into a strict M1/M2 dichotomy (43). In particular, Traeuble et al. generated a comprehensive atlas of human atherosclerotic plaques and identified multiple macrophage populations with specialized inflammatory, lipid-handling, and reparative functions. Among these are triggering receptor expressed on myeloid cells 2(TREM2) high lipid-associated macrophages (LAMs), which are enriched in advanced plaques and highly express genes involved in lipid metabolism and phagocytosis (44). These foam-cell-like macrophages may exert both protective and pathogenic roles depending on disease stage, contributing to lipid clearance and efferocytosis while also promoting necrotic core expansion when lipid accumulation becomes excessive. Another subset, folate receptor 2 (FOLR2) macrophages, exhibits tissue-resident and reparative characteristics, including extracellular matrix remodelling and anti-inflammatory activity (45). In contrast, interferon-responsive macrophages express interferon-induced transmembrane protein 3 (*IFITM3)*, interferon-stimulated gene 15 (*ISG15)*, interferon gamma-induced protein 10 (IP-10/ CXCL10), and other interferon-stimulated genes, reflecting activation by type I interferon signalling in inflammatory plaque environments. Additional populations identified by scRNA-seq include inflammatory IL1B macrophages, resident-like Lymphatic Vessel Endothelial Hyaluronan Receptor 1 (LYVE1) macrophages, and proliferating macrophages, collectively emphasizing the dynamic plasticity of macrophage phenotypes during plaque progression (46, 47).

Foam cell formation is a central event in atherosclerosis and results primarily from excessive macrophage uptake of ox-LDL via scavenger receptors such as CD36, SR-A1, and LOX-1 (48). When intracellular cholesterol accumulation exceeds the cell's efflux capacity, lipid droplets accumulate, and macrophages transform into foam cells, contributing to chronic inflammation, necrotic core formation, and plaque instability (49). Importantly, recent transcriptomic analyses indicate that foam cells are not merely passive lipid-storage cells but instead encompass diverse inflammatory, reparative, and metabolically adaptive phenotypes (50). Maintenance of intracellular cholesterol homeostasis depends largely on the cholesterol efflux transporters ATP-binding cassette transporter A1 (ABCA1) and ATP-binding cassette transporter G1 (ABCG1). ABCA1 mediates cholesterol efflux to apolipoprotein A-I (ApoA-I), whereas ABCG1 facilitates cholesterol export to mature high-density lipoprotein (HDL). Together, these transporters support reverse cholesterol transport and protect against foam cell formation (51). Deficiency of ABCA1 or ABCG1 in macrophages accelerates lipid accumulation, oxidative stress, inflammasome activation, and plaque progression, whereas increased expression of these transporters enhances cholesterol efflux, attenuates inflammatory signalling, and promotes plaque stabilization (52). Their expression is primarily regulated through liver X receptor (LXR) and retinoid X receptor (RXR) signalling pathways activated by intracellular oxysterols generated during cholesterol overload. Impaired ABCA1/ABCG1 function has also been associated with defective efferocytosis and increased macrophage apoptosis, thereby contributing to the expansion of the necrotic core and plaque vulnerability. Consequently, ABCA1- and ABCG1-mediated cholesterol efflux represents a major atheroprotective mechanism and an important therapeutic target in cardiovascular disease (53).

#### 3.1.2 Dendritic cells

Dendritic cells (DCs) occupy a pivotal position at the interface of innate and adaptive immunity in atherosclerosis, functioning as professional antigen-presenting cells that shape both local plaque inflammation and systemic immune responses. Under homeostatic conditions, DCs reside in the intima of large arteries, particularly at sites susceptible to disturbed hemodynamic flow, where they perform continuous immunosurveillance as vascular sentinels (54). In the setting of hypercholesterolemia and endothelial dysfunction, the intimal DC pool expands through local monocyte differentiation and *in situ* proliferation. These cells internalize modified lipoproteins, including ox-LDL, into lysosomal compartments and detect danger signals through pattern-recognition receptors such as TLRs and SRs, thereby amplifying vascular inflammation (55).

A central function of DCs in atherogenesis is processing and presenting lipid and protein antigens derived from ox-LDL and apoptotic cells. By presenting these antigens as peptide-MHC class II complexes to CD4⁺ T cells, DCs drive the preferential expansion of pro-atherogenic Th1 subsets, which in turn produce interferon-*γ* (IFN-*γ*) and potentiate macrophage activation within the plaque (39). A subset of DCs also migrates to para-aortic draining lymph nodes, where they contribute to systemic adaptive immune priming and the perpetuation of chronic vascular inflammation (56).

Single-cell RNA sequencing and fate-mapping studies have substantially revised our understanding of DC heterogeneity within atherosclerotic lesions (44). Three major subsets have been characterised: conventional DC type 1 (cDC1), which specialises in cross-presentation to CD8⁺ T cells and produces IL-12, thereby sustaining Th1-skewed responses; conventional DC type 2 (cDC2), which primarily activates CD4⁺ T helper cells and displays greater phenotypic plasticity; and plasmacytoid DCs (pDCs), which are potent producers of type I interferons (IFN-α/*β*) and have been detected in advanced human plaques where they may amplify innate inflammatory signals (42). In addition, single-cell atlases have identified TREM2⁺ lipid-associated macrophage/DC-like populations within plaques that exhibit features of both lineages and whose pro- or anti-atherogenic contributions remain an active area of investigation (44).

Importantly, not all DC subsets are pro-inflammatory. Tolerogenic DCs, characterized by low co-stimulatory molecule expression and high IL-10 production, can induce regulatory T cell (Treg) differentiation and promote immune homeostasis within the vessel wall (57). The balance between inflammatory and tolerogenic DC phenotypes is therefore a determinant of whether the plaque progresses or stabilizes. Beyond antigen presentation, activated DCs secrete cytokines, chemokines, and extracellular matrix-remodelling enzymes that enhance leukocyte recruitment and may weaken the fibrous cap, thereby contributing to plaque vulnerability (57).

The dual pro-inflammatory and tolerogenic capacity of DCs identifies them as attractive therapeutic targets. Experimental strategies under investigation include promoting tolerogenic DC differentiation with vitamin D receptor agonists and IL-10 supplementation, and selectively blocking DC-mediated co-stimulation to reduce pathogenic T cell activation, with the overarching aim of attenuating vascular inflammation without systemic immunosuppression (57).

#### 3.1.3 Neutrophils

Neutrophils are an essential component of the innate immune system in vertebrates because they represent the first line of defence against harmful bacteria that enter the body (58). They make up the majority of all white blood cells (WBCs), and because they don't live very long, they need to be replaced all the time to keep the quantity of them consistent (59). Neutrophils quickly stick to activated endothelium, move into tissues, eat pathogens, release antimicrobial proteins, and make reactive oxygen species (ROS) and neutrophil extracellular traps to get rid of microbes as quickly and effectively as possible when there is an infection or injury nearby (60).

The neutrophils, the most abundant circulating leukocytes, play an important role in sterile inflammation and injury in several ways. Recent data indicate a significant role for neutrophils in the pathogenesis of CAD, particularly during its subsequent consequences, including acute coronary syndrome (ACS) and heart failure (HF) (61).

It plays a role in both atherosclerosis and plaque destabilization. Neutrophils are disproportionately abundant in the vasculature of patients with unstable atherosclerotic plaques and correlate with histological characteristics indicative of plaque susceptibility (62).

Neutrophil extracellular traps (NETs), web-like structures composed of chromatin fibers and granular proteins released by activated neutrophils, have emerged as important mediators of atherosclerosis progression and plaque instability. NETs promote vascular inflammation by activating the NLRP3 inflammasome, inducing endothelial injury, and enhancing the recruitment of inflammatory cells within the arterial wall. In addition, NET formation contributes to the development of vulnerable plaques by stimulating oxidative stress, thrombosis, and endothelial dysfunction. Recent evidence has highlighted the critical role of NETs in plaque rupture and acute coronary events, suggesting that NET-associated pathways may represent potential therapeutic targets in cardiovascular disease (63, 64).

Both clinical and experimental research have conclusively confirmed the presence of neutrophils in early aortic lesions and in atherosclerotic plaques predisposed to rupture and erosion. Consequently, neutrophil count is regarded as an independent prognostic indicator in patients with acute coronary syndrome, both during admission and after revascularization (65).

### 3.2 Adaptive immunity

#### 3.2.1 T lymphocyte subsets

T lymphocytes are essential in both maintaining heart health and contributing to heart disease pathogenesis. The T cells encompass multiple distinct subtypes. There are two types of T lymphocytes: CD4 + and CD8 + . The CD4 + helper T (Th) cells can be categorized into four subtypes: Th1, Th2, Th17, and regulatory cells (Tregs) (51). Th1 cells secrete IFN-*γ* and protect the body against intracellular pathogens. Th2 cells produce IL-4 and IL-13, which promote allergic reactions and help fight helminth parasites. Th17 cells secrete IL-17 and take part in autoimmune and inflammatory conditions (66).

Tregs regulate immune tolerance by preventing the immune system from attacking the body's own tissue and limiting the occurrence of autoimmune diseases. Cytotoxic T cells, also known as CD8+ T cells, eliminate cells infected with viruses and those that have undergone cancerous transformation (67, 68). Additional subtypes include *γδ* T cells, which serve as a link between innate and adaptive immunity, and NK cells, which combine the characteristics of both NK cells and conventional T cells, enabling them to help control and respond effectively to the immune system (69).

Autoreactive CD4+ T-helper cells accumulate at the blood vessel wall during atherosclerosis-related inflammation. Apolipoprotein B (100) (apoB), the predominant protein component of low-density lipoproteins, serves as an autoantigen triggering pathogenic T-helper type 1 (TH1) cell-mediated secretion of pro-inflammatory cytokines. Clinical evidence supports the existence, within healthy humans, of a population of apoB-specific CD4+ T cells with an atheroprotective regulatory (Treg) phenotype. However, the role of apoB-reactive Tregs and their cross-talk with pathogenic TH1 cells is unclear (66, 70).

#### 3.2.2 B lymphocytes and humoral immunity

B cells are involved in atherosclerosis through both atheroprotective and pro-atherogenic pathways, depending on the subset. Despite their relative rarity in the plaque core, B cells play a significant role through humoral immunity and cellular cross-talk (71).

The basis for expression of a protective function is supported by the finding that B-cell deficiency enhances atherosclerosis in mice (72). A major part of this protection is thought to be mediated by B-1 cells, which are located in the peritoneal and pleural cavities and produce natural IgM antibodies (73). Such antibodies bind oxidation-specific epitopes (e.g., phosphorylcholine on ox-LDL and apoptotic cell membranes). Natural IgM targeting these antigens may promote their clearance, inhibit macrophage uptake of ox-LDL, and modify or reduce pro-inflammatory responses, thereby reducing lesion formation. Low concentrations of circulating IgM are also a clinical risk factor for CVD and a higher rate of subsequent cardiovascular events (74).

In contrast, B-2 cells (conventional B cells) are considered proatherogenic. The cells activate both B and T cells and participate in this complex immunological cascade through at least three pathways: 1) producing IgG antibodies that form proinflammatory immune complexes with antigens such as ox-LDL; 2) secreting IgE which activate mast cells and basophils - serum IgE levels correlate with greater severity of CAD and (3) acting antigen-presenting cells to promote pro-inflammatory T-helper (Th) cell responses, thereby bridging humoral with cellular adaptivity immunity (75).

The locational aspects of B-cell function are also important. Although sparse in the intimal plaque, B cells cluster with T cells and dendritic cells within TLOs located in the adventitia of advanced atherosclerotic arteries. These TLOs are secondary lymphoid tissues where antigen-driven B cell activation, clonal expansion, and differentiation into plasma cells occur, serving as an extramural focus for a continued atherogenic humoral response, including antibody and cytokine production that drive plaque inflammation from outside in (76, 77).

Overall, the impact of B cells on atherosclerosis is determined by an equilibrium between B-1 cell-mediated anti-atherogenic or natural IgM-dependent responses and pro-atherogenic or induced functions of disease-propelling B-2 cells (via IgG/IgE-induced T cell activation) (78). This divergence identifies B cells as an interesting target for therapeutic intervention, potentially to boost protective IgM or to suppress a detrimental B-2 cell response to preserve plaque stability (75).

## 4 Systemic and environmental modulators

**Figure 2:**
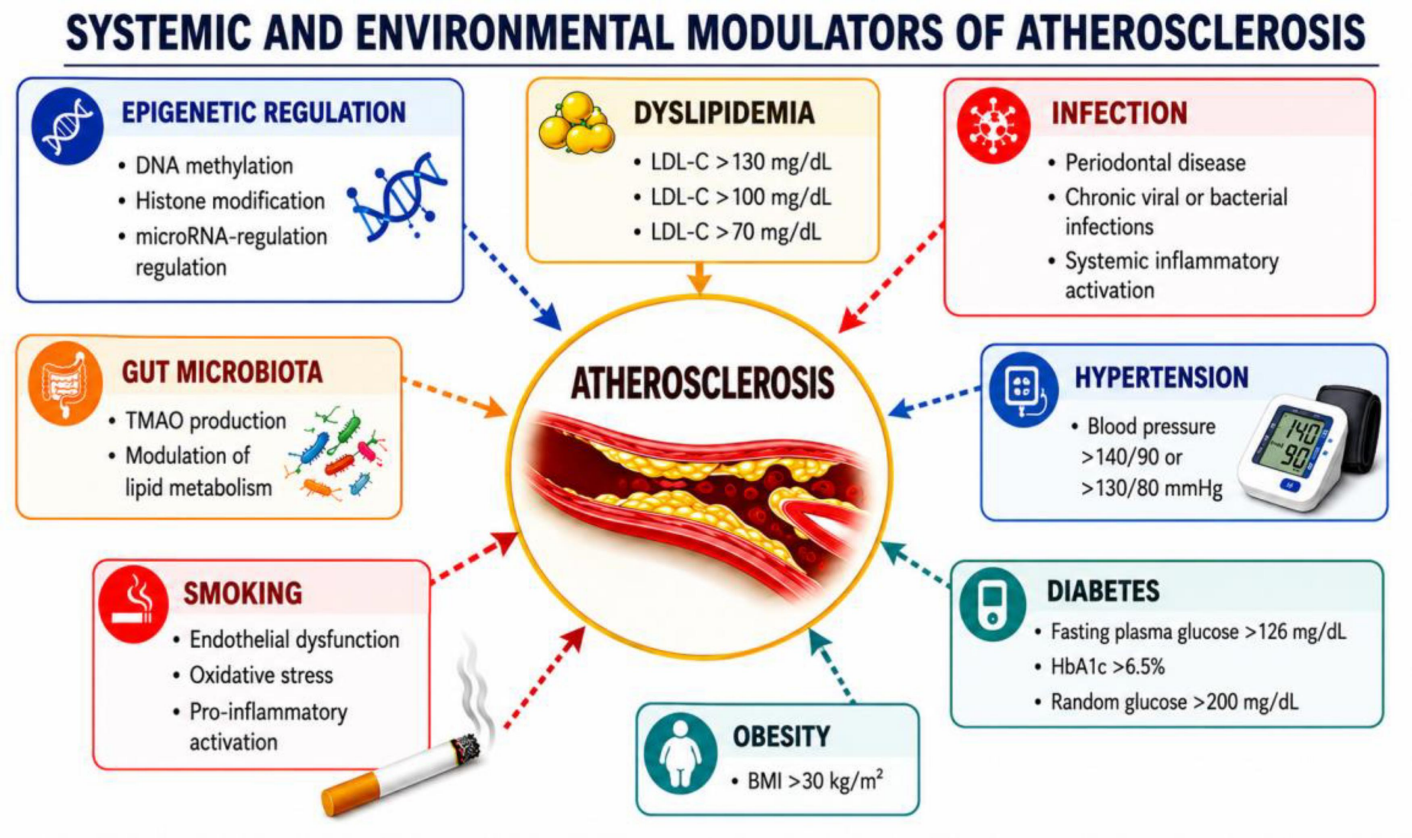
demonstrates that atherogenesis proceeds through multiple related mechanisms rather than a single linear pathway. Created with BioRender.com. in https://BioRender.com.

Systemic and environmental modulators of atherosclerosis are summarized in Figure 2.

### 4.1 Diabetes and hyperglycaemia

Diabetes is widely recognized as a chronic disease with a substantial health burden due to mortality or morbidity from the ultimate consequences of vascular complications (79). It was first observed as an independent cardiovascular disease risk factor in the Finnish study, demonstrating that type 2 diabetes conferred similar MI prognostic risk to those without type 2 diabetes, despite a prior Myocardial infarction (80).

Coronary artery disease is a major issue in diabetic individuals, whose inflammatory processes due to diabetes cause further narrowing of the coronary vessels and ultimately severe consequences such as death (81). The hyperglycaemia associated with diabetes can result in the modification of macromolecules, exemplified by the formation of advanced glycation end products (AGE). By binding to surface receptors such as RAGE (receptor for AGE), these AGE-modified proteins can enhance the production of proinflammatory cytokines and other inflammatory pathways in vascular endothelial cells. In addition to hyperglycaemia, diabetes induces oxidative stress through reactive oxygen species and carbonyl groups. Similar to hypertension, inflammation connects diabetes to atherosclerosis (82).

### 4.2 Obesity and adipokine dysregulation

The pathophysiological link connecting obesity to atherosclerosis has been significantly reinforced by epidemiological and experimental evidence over the past decades. Hence, at present, adipose tissue is not considered solely a storage tissue but rather an endocrine organ, exerting various physiological functions, regulating food intake, glucose and lipid metabolism, and thermogenesis, alongside neuroendocrine function, blood pressure, and immunity, through the release of mediators known as adipocytokines (83). In particular, visceral adipose tissue (VAT) plays a central role in the development of cardiometabolic disease. Expansion of VAT promotes adipose tissue dysfunction and chronic low-grade inflammation, characterized histologically by crown-like structures formed by macrophages surrounding necrotic adipocytes. This inflammatory milieu is associated with increased infiltration of pro-inflammatory M1 macrophages, which secrete cytokines including TNF-α, IL-6, and MCP-1. The persistent release of these mediators contributes to systemic meta-inflammation, endothelial dysfunction, and vascular injury, thereby accelerating atherogenesis (84).

Obesity not only predisposes individuals to insulin resistance and type 2 diabetes mellitus but also exacerbates atherogenic dyslipidaemia (85). Elevated concentrations of free fatty acids released from visceral fat enter the liver through the portal circulation, stimulating hepatic synthesis of triglyceride-rich VLDL. Increased VLDL levels enhance cholesteryl ester transfer protein (CETP)-mediated exchange of triglycerides and cholesterol esters between VLDL and HDL, thereby reducing HDL cholesterol concentrations. Furthermore, triglyceride enrichment of LDL particles promotes the formation of small dense LDL particles, which are more susceptible to oxidation, more readily penetrate the arterial intima, and are therefore considered highly atherogenic. Collectively, these mechanisms demonstrate that obesity independently promotes inflammation and atherosclerosis beyond its effects on insulin resistance and lipid metabolism alone (Table 2) (86).

**Table 2:** Modifiable and non-modifiable risk factors.

Non-modifiable risk factors	Genetics, Age, Gender, Family history of CVD, Previous history of CVD, Ethnicity/race
Lifestyle-associated risk factors	Smoking, Diet, Stress, physical inactivity, excessive alcohol consumption
Social risk factors	Social deprivation, Environment
Modifiable risk factors	Blood pressure, Cholesterol level, Blood glucose level, Body mass index
Outcome	Atherosclerosis → cardiovascular diseases

Source (129).

### 4.3 Hemodynamic factors: hypertension

Hypertension is most likely to affect the arterial tree through arterial wall thickening, the formation of atherosclerotic plaques, and increased susceptibility to plaque rupture. The vast majority of research, however, doesn't compare the impact of a wide range of BP values on the development of atherosclerosis (87).

The definitions of hypertension have differed between the American Heart Association/American College of Cardiology (AHA/ACC) and the European Society of Cardiology (ESC) guidelines since 2017. The 2017 ACC/AHA guidelines lowered the threshold and defined Stage 1 hypertension as SBP 130–139 mmHg or DBP 80–89 mmHg. In contrast, the ESC/ESH guidelines (including the 2023 ESC Guidelines on Cardiovascular Disease Prevention and the 2023 ESH Guidelines) maintained the diagnostic threshold for hypertension at office SBP ≥140 mmHg and/or DBP ≥90 mmHg; the range SBP 140–159 mmHg and/or DBP 90–99 mmHg corresponds to Grade 1 hypertension (88).

There is growing evidence that inflammation may contribute to hypertension, establishing a pathophysiological connection between these two conditions, similar to atherosclerosis. Angiotensin II (AII), in addition to its vasoconstrictor effects, can provoke intimal inflammation. For instance, AII stimulates arterial endothelial cells and SMCs to produce superoxide anion, a reactive oxygen species (89). AII can also enhance the expression of proinflammatory cytokines like IL–6, and MCP-1 by arterial SMCs, as well as the leukocyte adhesion molecule VCAM-1 on endothelial cells. Some of the clinical advantages of angiotensin-converting enzyme inhibitor therapy may result from the disruption of these proinflammatory pathways (90).

### 4.4 Epigenetic regulation and trained immunity

Epigenetic changes, such as DNA methylation, histone modifications, and chromatin remodelling, coordinate stimulus-specific transactivation of genes that promote atherosclerosis, integrating environmental and metabolic stress in the vasculature (91). These reversible heritable changes are adaptations that modulate the ability of all major vascular and immune cells, such as ECs and VSMCs, to respond to inflammatory, lipid-handling, and proliferative stimuli from macrophages or T lymphocytes (92).

Mechanistic Insights into Cellular Targets. In a pro-atherogenic setting, pro-inflammatory stimuli lead to hypermethylation of DNA at the promoters of protective genes. Indeed, certain negative features of the plaque have been shown to be possibly modulated by epigenetic control, such as the hypomethylation-mediated overexpression of inflammatory IL-1 and macrophage adhesion molecule VCAM-1 and the hypermethylation-driven silencing of anti-inflammatory IL-10 and NOS3 that decrease anti-inflammatory signalling and NO availability, which are expected to exacerbate endothelial dysfunction and plaque progression (93, 94).

On the other hand, gene expression programs for cholesterol efflux and inflammation resolution are repressed by higher expression of their antagonists, histone deacetylases (HDACs). Pharmacological classes 1 and 2 HDAC inhibitors (e.g., vorinostat, valproic acid) also prevent the repression of cholesterol efflux genes in preclinical studies, exerting anti-inflammatory and lipid-lowering effects, suggesting that epigenetic regulators are therapeutic targets for CVD (90).

Clinical evidence and systemic biomarkers indicate that epigenetic dysregulation is not confined to the plaque but also manifests systemically. For example, patients with angiographically proven CAD show increased genomic DNA methylation in lymphocytes compared to control subjects. Importantly, this hypermethylation is associated with elevated plasma homocysteine as an independent risk factor for CAD and predicts all-cause and cardiovascular mortality even in consideration of classical risk factors. Moreover, homocysteine levels might affect the methylation status of individual genes, including APOE, thereby linking a metabolic risk factor to epigenetic control of lipid metabolism. Although studies in Peripheral Blood Mononuclear Cell **(**PBMCs) reflect the systemic inflammatory environment, they highlight the importance of direct epigenetic profiling of vascular cells (ECs, VSMCs) within lesions to completely understand disease-associated cell-type-specific programs (95, 96).

The concept of trained immunity is an important contributor to the persistent inflammatory state in atherosclerosis. Trained immunity, as defined by Netea et al., refers to a form of non-specific innate immune memory mediated by epigenetic and metabolic reprogramming of monocytes and hematopoietic stem cells. This enables enhanced inflammatory responses upon re-exposure to stimuli, even in the absence of adaptive immune specificity. In the context of atherosclerosis, triggers such as oxidized LDL and hyperglycaemia can induce this trained phenotype in myeloid cells, sustaining chronic vascular inflammation that interacts with adaptive immune responses (97).

Therapeutic Implications and Future Directions. The plasticity of the epigenetic landscape provides a therapeutic opportunity unlike any other: to rewire pathological cellular phenotypes without changing DNA sequence. Contemporary approaches aim to reverse concrete pathological signs, for example, by up-regulating genes protective against disease (using HDAC inhibitors) or modulating pathways such as DNA methyltransferases. Potential future precision medicine strategies could include cell-specific epigenetic interventions designed to stabilize the plaque (e.g., prevent VSMC dedifferentiation, enhance macrophage cholesterol efflux) or reverse trained immunity induced by hematopoietic stem cells (93).

### 4.5 Microbiome and infection

Over the last decade, extensive research has examined the human microbiome and its role in mediating CVD (81). The complex, dynamic interactions between the microbiota and the host are being gradually deciphered by high-throughput technologies and multiomics analyses (82). Gut microbiome and CVD relationship have also been widely studied. The role of oral microbiome disruption (dysbiosis) is much less understood, though interest and the amount of literature evidence available from basic science, animal, and human studies in this research area are growing (98, 99).

Infectious agents may also provide inflammatory stimuli that enhance atherogenesis. Acute infections can modify hemodynamic and the coagulation and fibrinolytic systems, potentially leading to ischemic events (100).

Long-term extravascular infections, such as gingivitis, prostatitis, and bronchitis, can enhance the production of inflammatory cytokines in the extravascular space, potentially accelerating the progression of distant atherosclerotic lesions (101). Intravascular infection may potentially serve as a localized inflammatory stimulation that could expedite atherogenesis (102). Numerous human plaques exhibit indications of infection by microbial organisms such as Chlamydia pneumoniae (103). Chlamydia, when present in arterial plaque, may release lipopolysaccharide (endotoxin) and heat shock proteins that can stimulate the production of proinflammatory mediators by vascular endothelial cells, SMCs, and infiltrating leukocytes. Epidemiological studies of infection, however, have yielded mixed results, with limited prospective evidence indicating that antibodies directed against Chlamydia pneumoniae, Helicobacter pylori, herpes simplex virus, or cytomegalovirus predict vascular risk (104).

### 4.6 Clonal haematopoiesis of indeterminate potential (CHIP)

Clonal haematopoiesis of indeterminate potential (CHIP) is characterized by a clonally expanded haematopoietic cell lineage that carries a pathogenic mutation usually associated with hematologic cancers at a variant allele fraction of less than (VAF) ≥ 2% (90) in a person without a diagnosed hematologic malignancy or other clonal blood disorder (105, 106). Although CHIP is associated with more than a fourfold increased risk of hematologic malignancy, it has recently been recognized as an important measure for cardiometabolic health. Previous studies show that carriers of CHIP have an increased risk of CVD and mortality from all causes, with specific mutations associated with a higher CVD burden and larger clone sizes correlated with increased health burden (107). Functionally, macrophages lacking TET2 or DNA (Cytosine-5)-Methyltransferase 3 Alpha (DNMT3A) drive interleukin (IL)-1β/IL-6 inflammasome activation and promote atherosclerosis and metabolic dysregulation (108), while the Janus Kinase 2 Valine-to-Phenylalanine substitution at position 617F(JAK2V617F) mutation promotes thrombo-inflammatory phenotypes (109).

CHIP has also been linked with upstream metabolic precursors, including obesity and T2D, providing evidence for its involvement across the cardiometabolic disease continuum. Crucially, this relationship might be bidirectional: systemic inflammation drives the expansion of CHIP, and CHIP mutations accelerate inflammation. CHIP has been linked with early atherosclerosis with high variant allele fractions (VAF >10%) associated with greater coronary artery calcium scores CHIP mutations also increase the risk of coronary microvascular dysfunction in nonobstructive coronary artery disease CHIP was further implicated in incident coronary artery disease as demonstrated from previous work, notably by Jaiswal et al. showing a 1.9-fold higher risk in subjects without prior cardiovascular events for coronary artery disease (110). Importantly, JAK2V617F mutations conferred a 12-fold increased risk (111).

Recent progress has revealed insights into the mediators underlying the emergence of CHIP and pathways through which it promotes chronic diseases or worsens conditions (112). However, key questions remain, including how innate immune function is altered in CHIP and what signalling pathways underlie the links between inflammaging and the emergence and expansion of CHIP-mutant clones. From a medical perspective, it is important to identify which particular chronic diseases are at risk for developing when confronted with CHIP. Furthermore, do recipients of hematopoietic stem cell transplants hosting CHIP-mutant clones have increased risks for cardiometabolic or inflammatory disease (113).

## 5 Therapeutic implications and future directions

Atherosclerosis is an inflammatory, immune-mediated disease that has spurred numerous novel therapeutic approaches. Canakinumab (IL-1β inhibition) is among the current immune-targeting modalities for coronary vascular disease; in the CANTOS trial, it reduced cardiovascular event rates without lipid modification, albeit with an increased risk of fatal infection (114). In contrast, low-dose methotrexate showed no cardiovascular benefit in the CIRT trial, likely because it failed to lower IL-1β or IL-6 as anticipated (114, 115). Together, these outcomes highlight the challenges facing many immunomodulatory strategies. Notably, colchicine, via NLRP3 inflammasome inhibition, has since emerged as a clinically approved immunomodulatory therapy for CAD, based on the Low-Dose Colchicine2 (LoDoCo2) trial and the Colchicine Cardiovascular Outcomes (COLCOT) trial (116–118).

More recently, cardiometabolic therapies such as sodium–glucose cotransporter-2 (SGLT2) inhibitors and glucagon-like peptide-1 receptor agonists (GLP-1 RAs) have demonstrated anti-inflammatory and vasculoprotective effects beyond glycaemic control (119). Experimental and clinical studies suggest these agents attenuate atherosclerotic inflammation through mechanisms including suppression of the NLRP3 inflammasome, reduction of oxidative stress and endothelial dysfunction, modulation of macrophage polarization, and improvement in plaque stability (120). GLP-1 RAs appear particularly associated with reduced inflammatory cytokines and fewer atherosclerotic cardiovascular events, though disentangling their direct vascular effects from those mediated by weight loss and glycaemic improvement remains an active area of investigation (121). SGLT2 inhibitors have shown prominent benefits in heart failure and cardiorenal protection, with emerging evidence supporting their direct vascular anti-inflammatory actions. Recent data further suggest that combined SGLT2 inhibitor and GLP-1 RA therapy may exert additive cardiometabolic and anti-inflammatory benefits (122). In parallel, RNA-based lipid-lowering therapies such as inclisiran, a small interfering RNA (siRNA) targeting hepatic PCSK9 synthesis, have emerged as novel approaches for reducing atherosclerotic cardiovascular risk. Beyond potent and sustained LDL-cholesterol reduction, inclisiran may exert indirect anti-inflammatory and plaque-stabilizing effects by modulating lipid-driven vascular inflammation, although its immunomodulatory properties and long-term effects on plaque biology remain under investigation (123).

New therapeutic modalities include: (1) targeting specific immune cell subsets (Treg augmentation, B-2 cell inhibition); (2) using histone deacetylase (HDAC) inhibitors or DNA methyltransferase inhibitors to modulate epigenetic programs; (3) inhibiting inflammatory pathways in carriers of clonal haematopoiesis (CHIP); (4) targeted modulation of gut microbiota through dietary or pharmacologic intervention; and (5) vaccination against atherogenic antigens, such as apoB (107).

Future research should focus on defining the functional heterogeneity of plaque immune cells at single-cell resolution; elucidating how immune responses are spatially organized within plaques and in adventitial TLOs, identifying protective vs. pathogenic transcriptomic biomarkers to predict responses to specific immunomodulatory therapies; and developing cell-specific delivery systems for targeted intervention. In parallel, further mechanistic and translational studies are needed to clarify how emerging cardiometabolic agents, including SGLT2 inhibitors and GLP-1 receptor agonists, modulate vascular immune pathways and plaque biology independently of glucose lowering, and to determine whether these effects can be harnessed for precision anti-inflammatory therapy in atherosclerotic cardiovascular disease.

## 6 Conclusion

Atherosclerosis is a complex, multi-stage chronic inflammatory disease mediated by elaborate networks of innate and adaptive immune cells. The longstanding view of atherosclerosis as an inert lipid storage disease has been replaced by one that emphasizes endothelial activation, immune cell recruitment, and prolonged inflammation, which interact to drive plaque formation and progression. Single-cell RNA-seq technologies have transformed our understanding of heterogeneity within the immune compartment, uncovering surprising functional diversity within macrophage, T cell, and B cell subsets that cannot be easily categorized using binary classification systems.

Novel concepts such as VSMC phenotypic switching to macrophage-like cells, clonal haematopoiesis driving trained immunity, and the systemic effects of gut microbiota and epigenetic alterations have considerably broadened our cognitive window. These insights place the immune system at the forefront of atherosclerosis pathogenesis and underscore the link between local vascular inflammation, systemic metabolism, and environmental stimuli. The implications for therapy might be significant: targeting lipid-lowering beyond its own effects, while accounting for the broader audience constraints of targeted immunomodulation, could present an attractive proposition against residual cardiovascular risk. Important hurdles remain, however, including targeting cell-specific interventions vs. non-specific immunosuppression and identifying the population most likely to benefit from specific immunomodulatory strategies. As we continue to understand immune-cell granularity, the ability to leverage precision to tailor immunomodulation as a strategy for both prevention and treatment of atherosclerosis edges ever closer.
